# Intraoperative esketamine infusion and postoperative sleep quality after shoulder arthroscopy: a randomized controlled trial

**DOI:** 10.3389/fphar.2026.1864069

**Published:** 2026-07-01

**Authors:** Chaohui Liu, Wenying Lu, Lifeng Ni, Shengjie Yao

**Affiliations:** Hangzhou Normal University Affiliated Xiaoshan Hospital, Hangzhou, China

**Keywords:** esketamine, NMDA receptor antagonist, postoperative sleep disturbance, randomized controlled trial, shoulder arthroscopy, sleep quality

## Abstract

**Background:**

Sleep disturbance is common after shoulder arthroscopy and may impair postoperative recovery. Whether intraoperative esketamine infusion improves postoperative sleep quality in this population remains unclear.

**Methods:**

In this single-center, randomized, double-blind, controlled trial, adult patients with preoperative sleep disturbance undergoing shoulder arthroscopy were assigned (1:1) to receive intraoperative esketamine infusion (0.3 mg/kg/h) or placebo. The primary outcome was sleep disturbance (Athens Insomnia Scale [AIS] ≥6) on postoperative day 1 (POD1). Secondary outcomes included subjective and objective sleep parameters, pain scores, psychological outcomes, and adverse events.

**Results:**

Eighty patients were included. The incidence of sleep disturbance on POD1 was significantly lower in the esketamine group compared with the placebo group (5.0% vs. 27.5%; risk ratio 0.18, 95% confidence interval 0.04–0.77). Wearable-derived monitoring on POD1 showed a longer device-estimated total sleep time in the esketamine group (*P* < 0.001). Exploratory device-estimated sleep-stage parameters also favored the esketamine group (all *P* < 0.001). Subjective sleep measures also favored the esketamine group, which had lower Athens Insomnia Scale scores on POD1, POD3, and POD7 (all P < 0.05). At POD30, the PSQI score remained lower in the esketamine group than in the placebo group (5.5 (4.0–8.0) vs. 8.0 (6.0–8.3); P = 0.001).

**Conclusion:**

In this single-center randomized trial, intraoperative esketamine infusion was associated with reduced early postoperative sleep disturbance in patients undergoing shoulder arthroscopy. These preliminary findings warrant confirmation in larger multicenter trials.

**Clinical Trial Registration:**

MR-33-26-017245 (https://www.medicalresearch.org.cn/).

## Graphical Abstract

**Figure:**
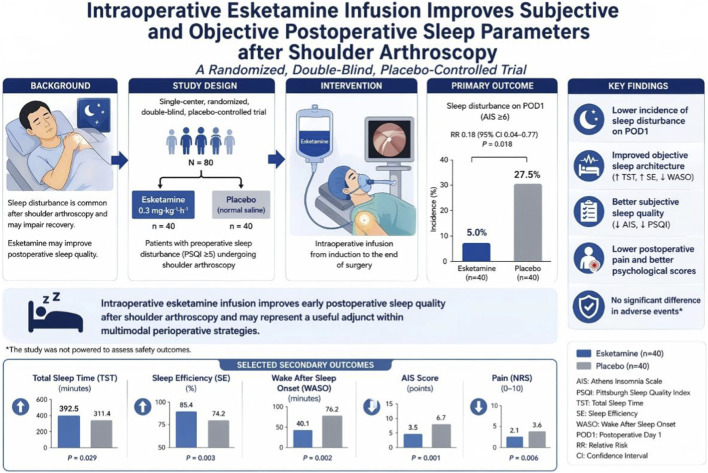

## Highlights

- Intraoperative esketamine infusion was associated with a lower incidence of early postoperative sleep disturbance after shoulder arthroscopy.
- Wearable-derived and subjective sleep outcomes favored the esketamine group during early postoperative recovery.
- The potential role of esketamine in perioperative sleep recovery requires confirmation in larger multicenter trials.

## Introduction

Postoperative sleep disturbance is a frequent yet underappreciated perioperative problem (Chen et al., 2025). It manifests as reduced sleep efficiency, sleep fragmentation, shortened rapid eye movement sleep, altered circadian rhythm, and poor subjective sleep quality (Zhao and Hu, 2025). These obstacles are of significant clinical importance. Postoperative sleep disorders can lead to cognitive dysfunction, delayed functional recovery, and cardiovascular events in patients (Zheng et al., 2023; da Silva et al., 2024; Pinilla et al., 2025). Recent evidence also indicates that sleep impairment is a modifiable risk factor linked to worse pain and greater analgesic requirements (Rouhi et al., 2023).

These issues are particularly pertinent to shoulder arthroscopy. Patients undergoing arthroscopic shoulder procedures, especially those with rotator cuff pathology, commonly experience marked preoperative sleep impairment because of nocturnal pain, difficulty finding a comfortable position, and limited ability to lie on the affected side (Hammad et al., 2022). Although arthroscopic shoulder surgery can effectively treat rotator cuff lesions, the incidence of postoperative sleep disorders is as high as 20%–40%, which may adversely affect postoperative rehabilitation and quality of life (Kunze et al., 2020).

Esketamine, the S-enantiomer of ketamine, has emerged as a potentially attractive adjunct in this context. In addition to its opioid-sparing analgesic properties, esketamine may influence affective symptoms, inflammatory responses, and sleep–wake regulation (Wei et al., 2022; Wen et al., 2024; Zhang et al., 2025). This multimodal profile is clinically appealing because postoperative sleep disturbance is itself multifactorial and cannot be fully explained by pain alone. Over the past few years, randomized trials in non-shoulder surgical populations have reported that intraoperative or perioperative esketamine may reduce the incidence of postoperative sleep disturbance and improve subjective sleep quality, including in gynecological laparoscopy, patients with pre-existing sleep disorders, and other general surgical settings (Song et al., 2025; Wu et al., 2025). A 2025 systematic review and meta-analysis pooling 1,287 patients further supported a beneficial overall effect of intraoperative esketamine on postoperative sleep quality (Zeng et al., 2026). Nevertheless, the current evidence base remains heterogeneous with respect to patient selection, type of surgery, dosing regimens, outcome definitions, and perioperative analgesic protocols. More importantly, procedure-specific evidence remains scarce, and findings from abdominal or gynecologic surgery cannot be directly extrapolated to shoulder arthroscopy, where sleep disruption is strongly shaped by positional discomfort, shoulder immobilization, and distinctive postoperative pain patterns.

Given these considerations, determining whether intraoperative esketamine improves postoperative sleep quality after shoulder arthroscopy is clinically meaningful. We therefore conducted a randomized controlled trial to assess the effect of intraoperative esketamine infusion on sleep quality in patients undergoing shoulder arthroscopic surgery. We hypothesized that, compared with placebo, intraoperative esketamine would improve early postoperative sleep quality and might also provide secondary benefits for pain and overall recovery.

## Methods

### Study design and ethics

This study was a single-center, prospective, randomized, double-blind, controlled trial. The study protocol was approved by the Institutional Medical Ethics Committee (Approval No.: [2025] 22) and registered with the China National Medical Research Registration and Filing Information System (Registration No.: MR-33-26-017245). Written informed consent was obtained from all participants or their legal representatives. The trial has been synchronized to the Chinese Clinical Trial Registry (ChiCTR), a WHO-recognized primary registry.

Patients scheduled for elective shoulder arthroscopy under general anesthesia between August 2025 and February 2026 were enrolled. Inclusion criteria were age 18–70 years, preoperative Pittsburgh Sleep Quality Index (PSQI) score ≥5, and American Society of Anesthesiologists (ASA) physical status classification I–III.

Exclusion criteria included severe cardiopulmonary disease, hypertension, history of intracranial hypertension, history of psychiatric disorders, communication barriers, long-term use of sedative-hypnotics or opioids, allergy to esketamine, cognitive impairments, hepatic or renal insufficiency, and severe obstructive sleep apnea syndrome requiring ventilator therapy. Criteria for withdrawal from the analysis included voluntary withdrawal by the participant, occurrence of severe intraoperative allergic reactions, and incomplete or missing data collection.

### Grouping and interventions

Eligible patients were randomly assigned in a 1:1 ratio to either the esketamine group or the placebo group using a random number table. The randomization sequence was generated by an independent researcher and concealed in sequentially numbered, sealed, opaque envelopes. These envelopes were opened only immediately before drug preparation by an independent anesthesia nurse who was not involved in any subsequent study procedures, including patient care, intraoperative management, postoperative follow-up, or data collection. The study drugs were prepared according to the randomization assignment: the esketamine group received 50 mg of esketamine diluted to 50 mL with 0.9% sodium chloride solution; the placebo group received 50 mL of 0.9% sodium chloride solution. The solutions for both groups were identical in appearance and volume. We selected an esketamine infusion rate of 0.3 mg/kg/h as a low-dose, subanesthetic intraoperative regimen based on prior perioperative studies of esketamine examining postoperative sleep disturbance and recovery (Qiu et al., 2022). The infusion began after anesthesia induction and was stopped 30 min before the anticipated end of surgery to minimize the risk of delayed emergence or early postoperative psychomimetic effects. Total esketamine dose was reported descriptively.

Patients, surgeons, anesthesiologists, and personnel responsible for postoperative follow-up and data collection were all blinded to group assignment. Unblinding was performed after completion of all data analyses.

### Anesthesia management protocol

All patients fasted preoperatively as per standard guidelines and received no premedication. Upon entering the operating room, patient identity was verified, baseline data were recorded, a peripheral intravenous line was established, and a multiparameter monitor was connected for continuous monitoring of electrocardiogram (ECG), heart rate (HR), pulse oxygen saturation (SpO_2_), and invasive arterial blood pressure (IBP). Preoperatively, an ultrasound-guided interscalene brachial plexus block was performed on the operative side using 0.25% ropivacaine (total volume 15 mL).

Anesthesia was induced with propofol 2 mg/kg, sufentanil 0.3 μg/kg, and rocuronium 0.6 mg/kg. Following endotracheal intubation, a continuous intravenous infusion was initiated at the beginning of the surgery. The esketamine group received esketamine at 0.3 mg/kg/h, while the placebo group received an equal volume and rate of 0.9% sodium chloride solution. The infusion was discontinued 30 min before the anticipated end of surgery. Anesthesia was maintained with 1.5% sevoflurane inhalation. The depth of anesthesia was adjusted based on the Bispectral Index (BIS, maintained between 40 and 60) and hemodynamic parameters. For the revised analysis, intraoperative BIS parameters were summarized, including mean BIS and the proportion of anesthetic time with BIS <40. Additional doses of rocuronium were administered as needed. At the end of surgery, ondansetron 4 mg was administered intravenously for prophylaxis of postoperative nausea and vomiting, and sugammadex 2 mg/kg was administered to reverse neuromuscular blockade. Postoperative analgesia was provided via patient-controlled intravenous analgesia (PCIA) with sufentanil 100 μg diluted to 100 mL with 0.9% sodium chloride solution. The PCIA settings were a background infusion at 1 mL/h, a bolus dose of 3 mL, and a lockout interval of 10 min. Postoperative analgesia was standardized for all patients. Rescue analgesia was allowed only when pain control remained inadequate despite PCIA, defined as VAS ≥4. The rescue regimen was the same in both groups and comprised an intravenous injection of parecoxib sodium 40 mg. The number of patients requiring rescue analgesia was recorded.

### Observation indicators

The primary outcome was the incidence of postoperative sleep disturbance on postoperative day 1 (POD1). Postoperative sleep disturbance on POD1 was assessed using the Athens Insomnia Scale (AIS) (Okajima et al., 2020; Jahrami et al., 2023). For postoperative assessments, the recall period was adapted to refer to the preceding postoperative night. Because the AIS was originally validated for assessing insomnia symptoms over a longer recall period, AIS ≥6 on POD1 was used as a pragmatic, trial-defined threshold for postoperative sleep disturbance rather than as a diagnostic cutoff for chronic insomnia.

Secondary outcomes included AIS scores on POD3 and POD7 and a PSQI score within 30 days postoperatively. Objective sleep parameters on POD1 were recorded using a wrist-worn wearable device (HUAWEI Band 10, Huawei, Shenzhen, China). The wearable collects physiological data via a green-light photoplethysmography (PPG) sensor (heart rate, HRV) and an accelerometer (body movement), processed by the embedded TruSleep™ 4.0 algorithm (Shang et al., 2024; Yang et al., 2025). Because wrist-worn wearable devices have limited accuracy for differentiating sleep stages compared with polysomnography, particularly for deep sleep and REM sleep, these variables were interpreted as device-estimated sleep parameters rather than definitive polysomnographic sleep-stage measurements. Although wearable devices have lower accuracy than polysomnography, previous validation studies have demonstrated acceptable agreement for estimating total sleep time and sleep efficiency, supporting their use in clinical research settings, although sleep staging remains less reliable (de Zambotti et al., 2015; 2019; Kahawage et al., 2020). Wearable recordings were included only when the device was worn throughout the predefined overnight recording period and provided at least 6 h of analyzable data. Recordings were excluded if there was evidence of device removal, insufficient signal quality, synchronization failure, low battery, or missing sleep-stage output. Data quality assessment and extraction were performed by investigators blinded to group allocation. These devices were used to estimate sleep-related parameters rather than to provide diagnostic sleep staging. Secondary outcomes also included anxiety and depression scores 1 day preoperatively and on POD1, POD3, and POD7; visual analog scale (VAS) scores at rest and during movement 1 day preoperatively and at 24 and 48 h postoperatively; total sufentanil consumption and number of PCIA demands at 24 and 48 h postoperatively; and the incidence of adverse events (including postoperative hypotension, delirium, nausea and vomiting, etc.). We additionally considered mild symptoms potentially related to esketamine exposure, including dizziness, blurred vision, abnormal dreams, euphoria, hallucination-like experience, and dissociative symptoms, when such information was available from clinical records and postoperative follow-up.

### Sample size calculation

The sample-size calculation was based on the primary outcome, namely, the incidence of sleep disturbance on POD1. Based on preliminary data, the expected incidence was 35% in the placebo group and 5% in the esketamine group. With a two-sided α of 0.05% and 90% power, 32 evaluable patients per group were required. To allow for a potential dropout rate of up to 25%, we planned to enroll up to 45 patients per group. During the actual study period, 82 eligible patients were randomized, and 80 completed the primary outcome assessment. Because the actual post-randomization attrition rate was only 2.4%, the final analyzed sample size of 40 patients per group exceeded the minimum required number of evaluable patients. A *post hoc* power calculation using the observed event rates of 27.5% and 5.0% showed an achieved power of approximately 83.2% for the primary endpoint.

### Statistical analysis

Data distribution and descriptive statistics were assessed. Normality of continuous variables was evaluated using the Shapiro-Wilk test and visual inspection of Q-Q plots. Normally distributed variables are presented as mean ± standard deviation (SD), while non-normally distributed variables are presented as median (interquartile range, or IQR). Categorical variables are presented as counts and percentages.

For the primary outcome, between-group comparisons were performed using the chi-square test or Fisher’s exact test, as appropriate. The effect size was reported as the risk ratio (RR) with its 95% confidence interval (CI). Given the limited number of primary outcome events, we did not fit a fully adjusted multivariable logistic regression with multiple covariates. The primary analysis used an unadjusted between-group comparison, reporting effect size as risk ratio and absolute risk difference with 95% confidence intervals. As an exploratory sensitivity analysis, we fitted a reduced logistic regression model adjusted only for age and preoperative PSQI score. For secondary outcomes, comparisons of continuous variables between groups were performed using the independent samples t-test for normally distributed data and the Mann-Whitney U test for non-normally distributed data. Categorical variables were analyzed using the chi-square test or Fisher’s exact test. Eligible patients were required to have preoperative poor sleep quality, defined as PSQI ≥5. Although this threshold was used to identify patients with preoperative sleep disturbance, baseline PSQI severity was further considered in an exploratory subgroup analysis using PSQI 5–9 and PSQI ≥10 categories. The primary outcome was tested as the single confirmatory endpoint. Analyses of secondary outcomes were exploratory. No formal adjustment for multiplicity was applied to secondary endpoints or repeated time-point comparisons; therefore, the corresponding P values should be interpreted as descriptive and hypothesis-generating. No imputation was performed for missing data in this study.

A complete-case approach was employed for all statistical analyses without missing data imputation. All 80 patients had complete valid data for all outcomes at their respective time points: AIS at POD1, POD3, and POD7; PSQI at POD30; wearable sleep monitoring at POD1; VAS pain scores at POD1 and POD3; and HADS at POD1, POD3, and POD7. No patients were lost to follow-up after POD1.

All analyses were performed using “R” (Version 4.2.2, http://www.R-project.org, The R Foundation) and the Free Statistics analysis platform (Version 1.9, Beijing, China, http://www.clinicalscientists.cn/freestatistics). A two-sided P value of less than 0.05 was considered statistically significant.

## Results

**FIGURE 1:**
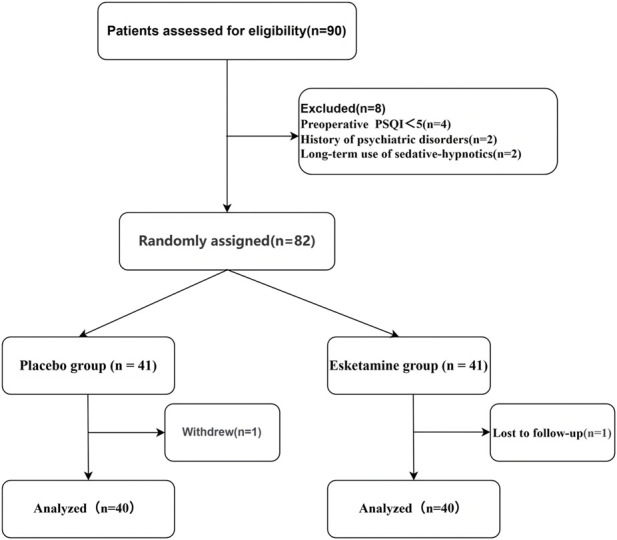
CONSORT flow diagram. PSQI, Pittsburgh Sleep Quality Index.

A total of 90 patients were assessed for eligibility. Eight patients were excluded before randomization, including four with a preoperative PSQI score <5, two with a history of psychiatric disorders, and two with long-term use of sedative-hypnotics. Eighty-two eligible patients were randomized in a 1:1 ratio, with 41 allocated to the esketamine group and 41 to the placebo group (Figure 1). After randomization, one patient in the placebo group withdrew consent and one patient in the esketamine group was lost to follow-up before completion of the primary outcome assessment. Finally, 80 patients were included in the primary analysis, with 40 patients in each group. The post-randomization attrition rate was 2.4%.

The baseline demographic and clinical characteristics were similar between the two groups. Shoulder impingement syndrome was numerically more frequent in the esketamine group than in the placebo group, although the difference did not reach conventional statistical significance. There were no differences in age, gender, BMI, comorbidities, preoperative sleep quality (PSQI), pain scores, or mood (all *P* > 0.05). No significant differences were observed in intraoperative BIS values between groups. Mean BIS was 49.0 ± 4.3 in the placebo group and 50.1 ± 4.2 in the esketamine group (P = 0.255). The proportion of anesthetic time with BIS <40 was 4.0% ± 0.7% and 4.1% ± 0.8%, respectively (P = 0.696). The requirement for rescue analgesia did not differ significantly between the placebo and esketamine groups (P = 0.645). Surgical and anesthesia-related variables were similar, indicating adequate baseline balance (Table 1).

**TABLE 1:** Baseline characteristics.

Characteristics	Total (n = 80)	Placebo group (n = 40)	Esketamine group (n = 40)	*P*
Age, years	58.3 ± 6.6	58.0 ± 5.9	58.6 ± 7.3	0.675
Male sex, n (%)	27 (33.8)	13 (32.5)	14 (35.0)	0.813
BMI, kg/m^2^	25.0 ± 3.1	24.9 ± 3.1	25.0 ± 3.1	0.908
Hypertension, n (%)	23 (28.7)	13 (32.5)	10 (25.0)	0.465
Diabetes, n (%)	9 (11.2)	6 (15.0)	3 (7.5)	0.481
Surgical diagnosis				
Rotator cuff injury, n (%)	58 (72.5)	28 (70.0)	30 (75.0)	0.617
Shoulder impingement syndrome, n (%)	37 (46.2)	14 (35.0)	23 (57.5)	0.054
Others, n (%)	13 (16.2)	8 (20.0)	5 (12.5)	0.363
Preoperative baseline				
PSQI	10.0 (8.0, 11.0)	10.0 (8.0, 11.0)	10.0 (8.0, 11.0)	0.551
Resting VAS	2.0 (2.0, 2.0)	2.0 (2.0, 2.0)	2.0 (2.0, 2.0)	0.822
Movement VAS	4.0 (4.0, 4.0)	4.0 (4.0, 4.0)	4.0 (4.0, 4.0)	0.230
HADS-A	5.0 (4.0, 6.0)	5.0 (4.0, 6.0)	5.0 (4.0, 5.2)	0.533
HADS-D	2.0 (2.0, 3.0)	2.0 (2.0, 3.0)	2.0 (1.0, 3.0)	0.686
Surgical characteristics				
Operative duration, min	93.8 ± 32.1	96.2 ± 32.0	91.3 ± 32.4	0.494
Anesthesia duration, min	134.2 ± 35.3	136.4 ± 37.1	131.9 ± 33.7	0.572
BIS	49.6 ± 4.3	49.0 ± 4.3	50.1 ± 4.2	0.255
Proportion of time with BIS <40, %	4.0 ± 0.7	4.0 ± 0.7	4.1 ± 0.8	0.696
Blood loss, mL	28.8 ± 15.8	31.5 ± 19.4	26.0 ± 10.6	0.120
Urine volume, mL	448.4 ± 247.0	460.5 ± 257.4	436.2 ± 238.9	0.664
IV fluid volume, mL	1,177.2 ± 336.4	1,218.2 ± 286.1	1,136.2 ± 379.4	0.278
Parecoxib sodium used, n (%)	5 (6.2)	3 (7.5)	2 (5.0)	0.645

Abbreviations: BMI, body mass index; PSQI, pittsburgh sleep quality index; VAS, visual analogue scale; HADS, hospital anxiety and depression scale; SD, standard deviation. Data are presented as mean ± SD, for normally distributed variables, median (IQR) for non-normally distributed variables, and n (%) for categorical variables. BIS, Bispectral Index. Surgical diagnoses were not mutually exclusive; some patients had more than one shoulder pathology, such as rotator cuff injury accompanied by shoulder impingement syndrome.

**FIGURE 2:**
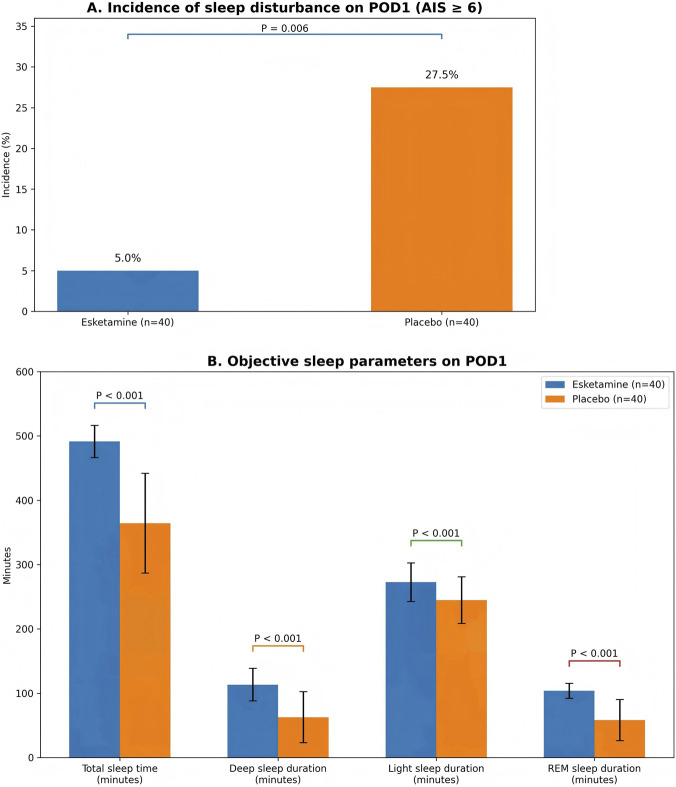
Primary and sleep-related outcomes. **(A)** Incidence (%) of postoperative sleep disturbance (AIS ≥6) on postoperative day 1 (POD1). **(B)** Objective sleep parameters on POD1. Data are presented as mean ± standard deviation (SD). Abbreviations: POD, postoperative day; REM, rapid eye movement. Values derived from the wrist-worn device are presented as wearable-derived estimates. Sleep-stage parameters were not confirmed by polysomnography and should therefore be interpreted as exploratory.

**FIGURE 3:**
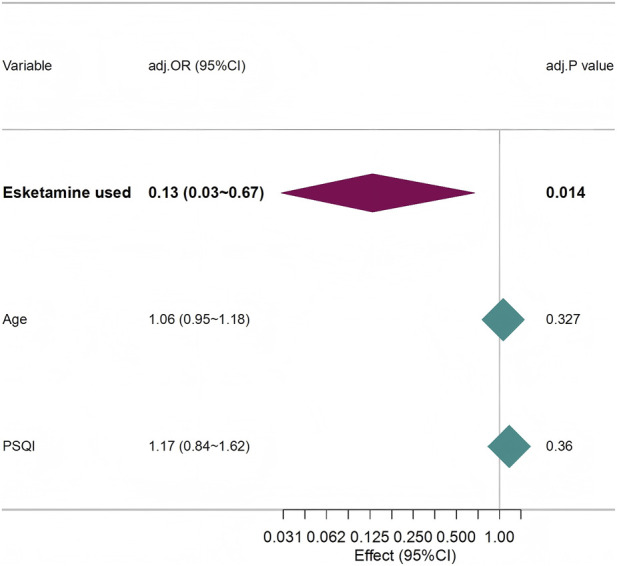
Exploratory reduced logistic regression analysis for postoperative sleep disturbance. Exploratory reduced logistic regression analysis for factors associated with postoperative sleep disturbance on POD1. This simplified sensitivity analysis was adjusted only for age and preoperative PSQI score. The original fully adjusted multivariable model was removed due to high risk of overfitting given the limited number of primary outcome events (n = 13). Intraoperative esketamine administration was associated with a reduced risk of postoperative sleep disturbance (OR 0.13, 95% CI, 0.03–0.67, P = 0.014). No significant associations were observed for age or preoperative PSQI score (all P > 0.05). All findings from this analysis are exploratory and should be interpreted cautiously.

The incidence of postoperative sleep disturbance on postoperative day 1 (POD1) was significantly lower in the esketamine group compared with the placebo group (5.0% vs. 27.5%; *P* = 0.006) (Figure 2). This corresponded to a risk ratio of 0.18 (95% CI, 0.04–0.77) and an absolute risk reduction of 22.5% (95% CI, 7.1%–37.9%) (Table 2). In an exploratory reduced logistic regression model adjusted only for age and preoperative PSQI score, the direction of the association remained consistent with the primary unadjusted analysis (P = 0.014) (Figure 3). To evaluate whether the effect of esketamine on postoperative sleep disturbance was modified by operative duration, we performed an exploratory interaction analysis using a multivariable logistic regression model adjusted for age and preoperative PSQI score. As shown in Supplementary Table S1, there was no significant interaction between treatment group and operative duration (OR 0.99, 95% CI 0.96–1.02, P = 0.482). There was no evidence that operative duration modified the association between esketamine infusion and reduced risk of early postoperative sleep disturbance.

**TABLE 2:** Sleep-related outcomes.

Outcome	Total (n = 80)	Placebo group (n = 40)	Esketamine group (n = 40)	Effect size (95% CI)	*P*
Primary outcome					
Sleep disturbance on POD1, n (%)	13 (16.2)	11 (27.5)	2 (5.0)	RR 0.18 (0.04–0.77) ARD −22.5% (−37.9% to −7.1%)	0.006
Objective sleep parameters (POD1)					
Total sleep time, min	427.8 ± 85.9	364.2 ± 77.6	491.4 ± 24.9	MD 127.2 (101.9–152.5)	<0.001

Data are presented as mean ± SD or number (percentage), unless otherwise indicated. Postoperative sleep disturbance was defined as an Athens Insomnia Scale (AIS) score ≥6. Risk ratio (RR) and absolute risk difference (ARD) with 95% confidence intervals (CIs) are reported for the primary outcome. Mean differences (MDs) with 95% CIs, are reported for continuous variables. RR, risk ratio; ARD, absolute risk difference; MD, mean difference; SD, standard deviation. Values derived from the wrist-worn device are presented as wearable-derived estimates.

On POD1, wrist-worn wearable monitoring showed a longer device-estimated total sleep time in the esketamine group than in the placebo group (Table 2). Device-estimated durations of deep sleep, light sleep, and REM sleep were also greater in the esketamine group; however, because sleep-stage classification from wrist-worn devices is less reliable than polysomnography-based staging, these findings are reported as exploratory wearable-derived estimates. Subjective sleep outcomes also favored the esketamine group, with lower AIS scores on POD1, POD3, and POD7 (all *P* < 0.05). At POD30, the PSQI score was lower in the esketamine group than in the placebo group (5.5 (4.0–8.0) vs. 8.0 (6.0–8.3); P = 0.001). These data are also provided in Supplementary Table S3.

Esketamine was also associated with lower postoperative pain scores and lower anxiety and depression scores during early recovery. No statistically significant difference in adverse events was observed between groups (Table 3).

**TABLE 3:** Clinically relevant adverse events.

Outcome	Total (n = 80)	Placebo group (n = 40)	Esketamine group (n = 40)	*P*
PACU				
Hypotension, n (%)	1 (1.3)	0 (0.0)	1 (2.5)	1.000
Nausea or vomiting, n (%)	5 (6.3)	2 (5.0)	3 (7.5)	1.000
Oversedation, n (%)	0 (0.0)	0 (0.0)	0 (0.0)	NA
POD1				
Hypotension, n (%)	0 (0.0)	0 (0.0)	0 (0.0)	NA
Nausea or vomiting, n (%)	9 (11.3)	3 (7.5)	6 (15.0)	0.675
Oversedation, n (%)	0 (0.0)	0 (0.0)	0 (0.0)	NA
Composite outcome				
Any adverse event, n (%)	9 (11.3)	3 (7.5)	6 (15.0)	0.481

PACU, post-anesthesia care unit; POD, postoperative day. Data are presented as n (%). P values were calculated using Fisher’s exact test. Any adverse event was defined as the occurrence of at least one of the following: hypotension, nausea or vomiting, or oversedation. NA, not applicable (no events occurred in either group).

Exploratory analyses of secondary outcomes suggested favorable trends in postoperative pain scores, psychological measures, and selected sleep-related parameters in the esketamine group. Because no multiplicity adjustment was applied, these secondary findings should be interpreted descriptively (Table 4). No severe psychomimetic adverse events, including hallucinations, agitation, or dissociation requiring clinical intervention, were observed. Mild psychomimetic symptoms were rare (Supplementary Table S4). Therefore, the possibility that subtle esketamine-related symptoms may have affected treatment perception cannot be excluded. In the esketamine group, the median total esketamine dose was 19.8 mg (IQR, 16.3–24.8 mg). Exploratory analyses did not show clear evidence that operative duration modified the treatment effect; however, these analyses were limited by the low number of primary outcome events. The enrolled cohort had a median baseline PSQI score of approximately 10, indicating that most participants had moderate-to-severe preoperative sleep disturbance. Exploratory subgroup analysis according to baseline PSQI severity showed directionally consistent findings; however, the analysis was underpowered and should be interpreted cautiously (Supplementary Table S5).

**TABLE 4:** Postoperative pain and psychological outcomes.

Outcome	Total (n = 80)	Placebo group (n = 40)	Esketamine group (n = 40)	*P*
Pain scores (VAS)				
Resting VAS, POD1	2.0 (1.0, 2.0)	2.0 (2.0, 2.0)	1.0 (1.0, 2.0)	<0.001
Movement VAS, POD1	3.0 (2.0, 4.0)	3.0 (3.0, 4.0)	2.0 (2.0, 3.0)	<0.001
Resting VAS, POD3	1.0 (0.0, 1.0)	1.0 (1.0, 1.0)	0.5 (0.0, 1.0)	0.009
Movement VAS, POD3	2.0 (1.0, 2.0)	2.0 (2.0, 2.0)	2.0 (1.0, 2.0)	0.015
Psychological outcomes (HADS)				
HADS-A, POD1	4.0 (3.0, 4.0)	4.0 (3.0, 5.0)	4.0 (3.0, 4.0)	0.041
HADS-D, POD1	2.0 (1.0, 2.0)	2.0 (2.0, 3.0)	2.0 (1.0, 2.0)	0.045
HADS-A, POD3	3.0 (3.0, 4.0)	4.0 (3.0, 4.0)	3.0 (3.0, 4.0)	0.008
HADS-D, POD3	2.0 (1.0, 2.0)	2.0 (1.0, 2.0)	1.0 (1.0, 2.0)	0.004

HADS, hospital anxiety and depression scale; POD, postoperative day. Data are presented as median (IQR) for continuous variables and n/N (%) for categorical variables. Secondary outcome analyses were exploratory, and P values are descriptive because no adjustment for multiple comparisons was performed. Median and IQR, values may include decimals because quartiles were calculated using interpolation.

## Discussion

This single-center randomized trial provides preliminary evidence that intraoperative esketamine infusion (0.3 mg/kg/h) may be associated with improved early postoperative sleep outcomes in patients with preoperative sleep disturbance undergoing shoulder arthroscopy. These findings are clinically relevant, given that postoperative sleep disturbance affects a substantial proportion of surgical patients and has been consistently linked to delayed functional recovery, increased pain sensitivity, and higher risk of adverse perioperative events (Rampes et al., 2019; Luo et al., 2020). Our results align with a growing body of literature suggesting that perioperative esketamine may improve postoperative sleep quality across diverse surgical populations (Qiu et al., 2022; Chen et al., 2025; Zhou et al., 2025). An exploratory reduced logistic regression model adjusted only for age and baseline PSQI score showed a directionally consistent association between esketamine and reduced postoperative sleep disturbance; however, this analysis was underpowered and should be interpreted cautiously. While these preliminary findings suggest that esketamine warrants further evaluation as part of multimodal perioperative care, larger multicenter trials are required to confirm the clinical benefit before widespread implementation.

The mechanisms underlying the observed association between intraoperative esketamine and improved early postoperative sleep are likely multifactorial and remain incompletely understood. Importantly, the concurrent improvements in postoperative pain control and reduced opioid consumption observed in the esketamine group should be interpreted not merely as separate secondary outcomes, but as plausible intermediate factors in the potential pathway linking esketamine exposure to improved sleep outcomes. Better analgesia may reduce nociceptive input, nocturnal arousal, and sleep fragmentation, while lower opioid requirements may minimize opioid-related disruption of sleep-wake regulation. In addition, the reduction in postoperative anxiety and depressive symptoms observed in this study is consistent with the known psychotropic profile of esketamine and could have contributed to improved perceived sleep quality by attenuating perioperative stress responses (Zhang et al., 2022; Mion and Himmelseher, 2024; Levinstein et al., 2025). Beyond these clinical pathways, esketamine may modulate glutamatergic neurotransmission, perioperative stress responses, and downstream neuroplastic processes, all of which are biologically linked to arousal regulation and circadian homeostasis (Dell'Osso and Martinotti, 2023; Guo et al., 2023; Zhang et al., 2024; Yuan et al., 2025). Prior preclinical and clinical studies of ketamine have also demonstrated effects on sleep-related neurophysiology, providing a plausible biological basis for the observed improvements in wearable-derived sleep parameters (Shen et al., 2024; Fountoulakis et al., 2025). However, the present study was not designed to formally test mediation or investigate neurobiological mechanisms directly, and the relationships among pain, opioid use, psychological symptoms, and sleep are likely bidirectional. Therefore, all mechanistic interpretations presented here should be regarded as plausible hypotheses rather than definitive conclusions.

Several limitations should be considered. First, objective sleep measures were obtained from a wrist-worn wearable rather than polysomnography. Although such devices can provide reasonable estimates of total sleep time in clinical research, their accuracy for classifying sleep stages is limited. These exploratory findings require confirmation in future studies employing polysomnography or other validated sleep-monitoring methods. Second, objective sleep monitoring was performed only on POD1, and therefore represented acute first-night postoperative sleep estimates rather than longitudinal objective sleep recovery. Future studies should incorporate multi-night objective sleep assessment to better characterize the overall recovery. Third, sleep outcomes were assessed primarily during the early postoperative period, and the longer-term durability of these benefits remains uncertain. In addition, no statistically significant difference in adverse events was observed between groups; however, the study was not powered to detect differences in safety outcomes or uncommon adverse events. Accordingly, these findings should not be interpreted as establishing safety equivalence. **F**urthermore, existing evidence remains heterogeneous with respect to patient populations, surgical procedures, and esketamine dosing strategies (Wang et al., 2025). Although the study was powered for the primary endpoint, the modest sample size reduced effect estimate precision and restrict cross-setting generalizability, as single-center trials tend to overstate therapeutic benefits. Accordingly, the observed reduction in postoperative sleep disturbance is preliminary and requires verification via large multicenter randomized trials.

Future large-scale, multicenter randomized trials are needed to confirm these preliminary findings. Such trials should enroll a broader spectrum of patients with varying severities of preoperative sleep disturbance, incorporate repeated objective sleep assessment using validated methods such as polysomnography or research-grade actigraphy, extend follow-up to evaluate long-term functional outcomes, and compare different esketamine dosing regimens to define the optimal strategy for improving postoperative sleep. Additionally, studies incorporating prespecified mediation analyses and mechanistic endpoints are required to clarify the biological pathways underlying the observed effects of esketamine on postoperative sleep recovery.

## Conclusion

In this single-center randomized trial, intraoperative esketamine infusion was associated with a lower incidence of early postoperative sleep disturbance after shoulder arthroscopy.

## Data Availability

The raw data supporting the conclusions of this article will be made available by the authors, without undue reservation.
