# Supplementary material for: Intraoperative esketamine infusion and postoperative sleep quality after shoulder arthroscopy: a randomized controlled trial
Source: Front Pharmacol. 2026 Jul 1;17:1864069. doi: 10.3389/fphar.2026.1864069 (PMC13368792; doi:10.3389/fphar.2026.1864069)
Supplement: Supplementary file 1 [file Table1.DOCX]

**Supplementary Table S1:Interaction analysis between treatment group and operative duration for postoperative sleep disturbance on POD1**

| Variable | β (SE) | Odds Ratio (OR) | 95% Confidence Interval | P value |
| --- | --- | --- | --- | --- |
| **Main effects** |  |  |  |  |
| Treatment group (Esketamine vs. Placebo) | -2.04 (0.83) | 0.13 | 0.03–0.67 | 0.014 |
| Operative duration (per 10-minute increase) | 0.00 (0.02) | 1 | 0.97–1.03 | 0.987 |
| **Interaction effect** |  |  |  |  |
| Treatment group × Operative duration | -0.01 (0.01) | 0.99 | 0.96–1.02 | 0.482 |
| **Adjusted covariates** |  |  |  |  |
| Age (per 1-year increase) | 0.02 (0.03) | 1.02 | 0.96–1.08 | 0.534 |
| Preoperative PSQI score (per 1-point increase) | 0.11 (0.12) | 1.12 | 0.89–1.41 | 0.337 |

Results are from a multivariable logistic regression model with postoperative sleep disturbance (AIS ≥ 6 on POD1) as the dependent variable. reatment group was coded as 0 = placebo group, 1 = esketamine group. Operative duration was treated as a continuous variable (unit: minutes). All P values are two-sided. This analysis was exploratory and not prespecified in the original study protocol. Results should be interpreted cautiously.

**Supplementary Table S2:Objective sleep parameters (POD1)**

| **Outcome** | **Total (n=80)** | **Placebo group(n=40)** | **Esketamine group (n=40)** | **Effect Size (95% CI)** | ***P*** |
| --- | --- | --- | --- | --- | --- |
| **Objective sleep parameters (POD1)** |  |  |  |  |  |
| Deep sleep duration, min | 88.0 ± 41.8 | 62.6 ± 39.7 | 113.4 ± 25.3 | MD 50.8 (36.2–65.4) | <0.001 |
| Light sleep duration, min | 258.7 ± 35.8 | 244.8 ± 36.2 | 272.6 ± 29.9 | MD 27.8 (13.3–42.3) | <0.001 |
| REM sleep duration, min | 81.1 ± 33.1 | 58.4 ± 32.0 | 103.8 ± 11.6 | MD 45.4 (34.9–55.9) | <0.001 |

Abbreviations: POD1, postoperative day 1; MD, mean difference; CI, confidence interval; REM, rapid eye movement sleep.Notes: Continuous variables are expressed as mean ± standard deviation. Intergroup comparisons were performed via independent-samples Student’s t-test. Mean difference (MD) and its corresponding 95% CI were reported as the between-group effect size. Eighty enrolled patients were randomly assigned equally into placebo group (n = 40) and esketamine group (n = 40).

**Supplementary Table S3: Sleep Quality**

| **Outcome** | **Control group**  **(n = 40)** | **Esketamine group**  **(n = 40)** | ***p*** |
| --- | --- | --- | --- |
| Subjective sleep quality |  |  |  |
| AIS on POD1 | 5.0 (3.8–6.0) | 3.0 (2.0–3.0) | <0.001 |
| AIS on POD3 | 4.0 (3.0–5.0) | 2.0 (1.0–2.3) | <0.001 |
| AIS on POD7 | 3.0 (2.0–4.0) | 2.0 (1.0–3.0) | <0.001 |
| PSQI on POD30 | 8.0 (6.0–8.3) | 5.5 (4.0–8.0) | 0.001 |

POD, postoperative day; AIS, Athens Insomnia Scale; PSQI, Pittsburgh Sleep Quality Index.Values derived from the wrist-worn device are presented as wearable-derived estimates. Sleep-stage parameters were not confirmed by polysomnography and should therefore be interpreted as exploratory.

# **Supplementary Table S4:** Clinically relevant adverse events

| **Outcome** | **Total (n = 80)** | **Placebo group**  **(n = 40)** | **Esketamine group**  **(n = 40)** | ***p*** |
| --- | --- | --- | --- | --- |
| Dizziness n (%) | 1(1.25) | 0 (0.0%) | 1 (2.5%) | 1.000 |
| Abnormal dreams, n (%) | 0 (0.0%) | 0 (0.0%) | 0 (0.0%) | NA |
| Hallucinations, n (%) | 0 (0.0%) | 0 (0.0%) | 0 (0.0%) | NA |
| Agitation, n (%) | 0 (0.0%) | 0 (0.0%) | 0 (0.0%) | NA |
| Dissociation, n (%) | 0 (0.0%) | 0 (0.0%) | 0 (0.0%) | NA |

NA, not applicable.Notes: Results are shown as n (%). Fisher’s exact test was used for between-group comparison; NA indicates unavailable P value due to zero adverse events in both arms. Each treatment arm included 40 patients.

**Supplementary Table S5: subgroup analysis**

| **Subgroup** | **Variable** | **n.total** | **n.event_%** | **crude.OR_95CI** | **crude.P_value** | **P.for.interaction_1** | **P.for.interaction_2** |
| --- | --- | --- | --- | --- | --- | --- | --- |
| **PSQI＜10** |  |  |  |  |  | 0.955 | 0.955 |
|  | **Placebo group** | 17 | 5 (29.4) | 1(Ref) | - |  |  |
|  | **Esketamine group** | 16 | 0 (0) | 0 (0~Inf) | 0.993 |  |  |
| **PSQI≥10** |  |  |  |  |  |  |  |
|  | **Placebo group** | 23 | 6 (26.1) | 1(Ref) | - |  |  |
|  | **Esketamine group** | 24 | 2 (8.3) | 0.22 (0.04~1.17) | 0.075 |  |  |
| **No Rotator cuff injury** |  |  |  |  |  | 0.475 | 0.994 |
|  | **Placebo group** | 12 | 2 (16.7) | 1(Ref) | - |  |  |
|  | **Esketamine group** | 10 | 1 (10.0) | 1.80 (0.15~22.3) | 0.997 |  |  |
| **Rotator cuff injury** |  |  |  |  |  |  |  |
|  | **Placebo group** | 28 | 8 (28.6) | 1(Ref) | - |  |  |
|  | **Esketamine group** | 30 | 2 (6.7) | 0.15 (0.03~0.78) | 0.054 |  |  |

PSD, postoperative surgical delirium; PSQI, Pittsburgh Sleep Quality Index; OR, odds ratio; CI, confidence interval; Ref, reference (placebo) group.Notes: Subgroup stratification was performed according to preoperative PSQI level (PSQI＜10 vs PSQI≥10) and the presence or absence of preoperative rotator cuff injury. Crude OR and corresponding 95% CIs were generated from unadjusted univariable logistic regression. P.for.interaction_1 was determined by likelihood ratio test to assess across-subgroup interaction; P.for.interaction_2 was obtained by adding the product interaction term of treatment and stratification factor into regression models. All statistical tests were two-sided.
